# 17beta-estradiol suppresses TLR3-induced cytokine and chemokine production in endometrial epithelial cells

**DOI:** 10.1186/1477-7827-3-74

**Published:** 2005-12-29

**Authors:** Margaret J Lesmeister, Rebecca L Jorgenson, Steven L Young, Michael L Misfeldt

**Affiliations:** 1 Department of Molecular Microbiology and Immunology, University of Missouri-Columbia, School of Medicine, Columbia, MO, USA; 2 Department of Obstetrics and Gynecology, University of North Carolina Medical School, School of Medicine, Chapel Hill, NC, USA

## Abstract

**Background:**

The human endometrium is an important site for contact between the host and pathogens ascending the reproductive tract, and thus plays an important role in female reproductive tract immunity. Previous work in our laboratory has suggested that Toll-like receptors (TLRs) are involved in endometrial epithelial recognition of pathogens and that ligation of endometrial TLRs results in the production of cytokines and chemokines important for both immune and reproductive functions of the endometrium. We have also demonstrated cyclic regulation of TLR3 mRNA and protein expression in human endometrium, suggesting that steroid hormones might play a role in the expression and function of TLR3. In this study, the effects of 17beta-estradiol (E2) and progesterone (P) on TLR3 expression and function in endometrial cell lines were investigated.

**Methods:**

Endometrial epithelial cell lines were cultured and examined for the presence of TLR3 and hormone receptors by endpoint RT-PCR. For hormonal studies, cells were pre-treated with ethanol vehicle, 10^(-8) M E2, and/or 10^(-7) M P. For antagonist assays, cells were treated with the ER antagonist, ICI 182, 780, or the PR antagonist, RU486, for two hours prior to treatment with hormones. Following hormone or hormone/antagonist pre-treatment, cells were stimulated with vehicle, the synthetic TLR3 ligand, polyinosinic-polycytidylic acid (Poly I:C), a negative dsDNA control, or a positive control. Cytokine and chemokine production post-stimulation was measured by ELISA. The effects of E2 and P on TLR3 mRNA and protein expression were measured using Real Time RT-PCR and FACS analysis, respectively.

**Results:**

Stimulation of TLR3-expressing cells with the synthetic TLR3 ligand, Poly I:C, resulted in the production of cytokines and chemokines important for endometrial function and regulation. Suppression of Poly I:C-induced cytokine and chemokine production by cells treated with 10^(-8) M E2, but not cells treated with 10^(-7) M P, was observed in endometrial epithelial cell lines expressing TLR3 and estrogen receptor alpha (ERalpha). The effects of E2 were not observed on cells which did not express ERalpha or in cells pre-treated with the ER antagonist, ICI 182, 780. Treatment with E2 did not affect TLR3 mRNA or protein expression. However, treatment with E2 did suppress cytokine and chemokine production resulting from TLR3 stimulation with Poly I:C, suggesting that E2 modulates TLR3 function.

**Conclusion:**

The data presented in this study are the first indication that E2 can markedly alter the innate immune response to dsRNA, providing a previously unreported process by which E2 can alter immune responses.

## Background

The human endometrium coordinates the reproductive events leading to embryo implantation and pregnancy. The surface and glandular epithelium of the endometrium is an important site of contact between the host and several pathogens ascending the reproductive tract, including gonorrhea, chlamydia, human immunodeficiency virus (HIV), cytomegalovirus (CMV), and herpes simplex virus (HSV), as well as allogeneic sperm and the semi-allogeneic embryo. Thus, the endometrial epithelium must tolerate contact with sperm and tissue invasion by the embryo, yet actively mount immune responses to pathogens in order to prevent infection.

A component of the endometrial epithelial response to pathogens is thought to be the elaboration of cytokines, which can activate both innate and acquired immune responses. Cytokines also play an essential role in regulating normal endometrial functions including embryo implantation, epithelial proliferation and shedding, and regulation of steroid hormone production[1-4]. The endometrial epithelium and stroma are rich sources of cytokine expression and important targets for cytokine action[1]. The importance of cytokines in the endometrium is further exemplified by the association between abnormal cytokine expression and endometrial dysfunctions including infertility, recurrent miscarriage, and endometriosis[1,5,6]. For example, Interleukin-6 (IL-6) and Interleukin-8 (IL-8) have been shown to be elevated in the peritoneal fluid of women with endometriosis, but the reason for this abnormal cytokine expression has not been determined [7-10].

Cyclic changes in endometrial cytokine expression suggest modulation of cytokine expression by estradiol (E_2_) and progesterone (P)[3,11]. *In vitro *studies have shown that E_2 _and/or P can either inhibit or stimulate expression of specific cytokines. Specifically, Pottratz and colleagues demonstrated suppression of cytokine-stimulated IL-6 mRNA by E_2 _in HeLa cells transfected with estrogen receptor (ER)[12]. Suppression of IL-6 was also observed by Tabibzadeh and colleagues in IL-1α-induced stromal cells[13]. Girasole and colleagues have demonstrated similar results using E_2 _on mouse cell lines and stromal cell lines[14]. P, at high concentrations, has been shown by Kelly and colleagues to reduce the level of IL-8 in the endometrium[3]. However, Tseng and colleagues found IL-6 to be up-regulated by E_2 _in stromal cells[15], while von Wolff and colleagues and Rifas and colleagues suggested hormones do not regulate IL-6[16,17]. Thus, studies examining the influence of steroid hormones on cytokine expression and production in the human have been somewhat conflicting and the impact of cytokine control by steroid hormones on mucosal immunity in the endometrium has not been elucidated[3,12-17]. The observed differences in the influence of E_2 _on cytokine expression may depend, in part, on the ligand-receptor signaling system responsible for the induction of cytokine expression and production.

Toll-like Receptors (TLRs) play an important role in recognition of pathogens and induction of several gene expression patterns during infection. TLRs are innate pattern recognition receptors (PRRs) characterized by amino-terminal leucine-rich repeats (LRRs) and carboxy-terminal Toll/IL-1 receptor (TIR) signaling domains [18-20] (for review see 20 and 21). TLRs recognize unique pathogen associated molecular patterns (PAMPs) to initiate and shape adaptive immune responses [21-24]. For example, TLR3 has been shown to bind double-stranded RNA (dsRNA), a product of most viral life cycles, and TLR4 recognizes bacterial lipopolysaccharide (LPS). TLR expression in the endometrial epithelium has been documented and endometrial TLRs, such as TLR3, have been shown to produce cytokines and chemokines including IL-6 and IL-8 upon TLR ligation [25-28]. Therefore, since TLR activation induces cytokine production and cytokines are required for proper endometrial function, TLRs may play a significant role in influencing and shaping immunological outcomes in the endometrium.

We and others have previously demonstrated expression of TLR3 mRNA and protein in the endometrium [25-28]. We also showed that TLR3 in the endometrium responded to dsRNA by producing specific cytokines and chemokines[26]. Although double-stranded RNA-activated protein kinase (PKR) has been shown to bind dsRNA, we found that the response to dsRNA in the endometrial epithelial cell line, RL95-2, was dependent on TLR3 by utilizing small inhibitory RNAs against TLR3[26]. Our findings established a requirement for TLR3 in the response to dsRNA in the RL95-2 cells, eliminating the possibility that PKR is responsible for the dsRNA response in endometrial epithelial cells[26]. Endometrial TLR3 expression has also been demonstrated to be cycle-dependent, with an approximately ten-fold up-regulation of TLR3 mRNA and protein during the mid- and late secretory phase of the menstrual cycle[26]. However, the role of steroid hormones in the regulation of TLR3 expression and/or function has not been examined. In this study, we investigated the role of E_2 _and P in the modulation of endometrial TLR3 expression and function.

## Methods

### Cell lines and cell culture

The endometrial epithelial cell lines, AN3-CA, HEC-1-A, KLE, and RL95-2, and the breast adenocarcinoma cell line, MCF7, were obtained from the American Type Culture Collection (ATCC) (Manassas, VA). KLE and RL95-2 cells were maintained in phenol red-free DMEM-F12, HEC-1-A cells in McCoy's 5a, AN3-CA cells in MEM, and MCF7 cells in EMEM. AN3-CA, HEC-1-A, KLE, and RL95-2 cells were supplemented with 2 mM L-glutamate and 50 μg/ml gentamicin (American Pharmaceutical Partners, Inc., Schaumburg, IL). MCF7 cells were additionally supplemented with 1 mM sodium pyruvate, 1 mM non-essential amino acids (NEAA), and 0.15 mg/ml sodium bicarbonate. All media contained 5% fetal bovine serum (FBS) (US Bio-Technologies, Inc., Parkerfield, PA). Forty-eight hours prior to experiments, media was replaced with CD-F12, a phenol red-free DMEM/F12 containing charcoal/dextran treated FBS (Hyclone, Logan, UT) and the previously mentioned additives specific for each cell line. Adherent cell lines were harvested using 0.05% trypsin/0.53 mM EDTA in HBSS. Unless otherwise indicated, all reagents were obtained from Invitrogen (Carlsbad, CA).

### Hormone treatment and cell stimulation

17β-estradiol (E_2_) and progesterone (P) (Calbiochem, San Diego, CA) were resuspended in ethanol to make a 1 mM stock solution of E_2 _and P. Prior to experiments, E_2 _and P stock solutions were diluted to the indicated concentrations using CD-F12. E_2 _and P concentrations were determined by performing a dose response. The chosen concentrations demonstrated the highest level of cytokine and chemokine suppression after stimulation, based on preliminary experiments, and were also physiologically relevant. Cells were examined for presence of hormone receptors by endpoint RT-PCR using primers listed in Table 1 prior to performing each experiment. Following 48 hours culture in CD-F12, cells were plated at 0.2 × 10^6 ^cells/well/ml in 12 well plates. The following day (~12 hours), CD-F12 media was aspirated and replaced with CD-F12 media containing either 0.001% ethanol vehicle (V), 10^-8 ^M E_2_, 10^-7 ^M P, or a combination of 10^-8 ^M E_2 _and 10^-7 ^M P. Triplicate wells were used for each hormone treatment condition and stimulation condition. For hormone receptor antagonist experiments, cells were pretreated for 2 hours with CD-F12 media containing V, 10^-6 ^M ICI 182, 780 or 10^-6 ^M RU486 (Tocris, St. Louis, MO). Following antagonist treatment, media was aspirated and replaced with CD-F12 containing V, 10^-8 ^M E_2_, or 10^-7 ^M P, with antagonist vehicle, 10^-6 ^M ICI 182, 780, or 10^-6 ^M RU486. Hormone and antagonist combinations are indicated for in each figure demonstrating hormone and/or antagonist results. Triplicate wells were used for each condition. After 48 hours treatment, the triplicate wells were stimulated with ligand. Supernatants and/or cells for each well were harvested after 18 hours of stimulation. Cells were stimulated with the synthetic TLR3 ligand, polyinosinic-polycytidylic acid (Poly I:C, 10 μg/ml) (Amersham Pharmaceutical Biotech, Piscataway, NJ), the double-stranded DNA negative control, polydeoxyinosinic-deoxycytidylic acid (Poly dI:dC, 10 μg/ml) (Amersham Pharmaceutical Biotech, Piscataway, NJ), or the positive control, phorbol-12-myristate-13-acetate (Calbiochem, San Diego, CA) plus Ionomycin (Calbiochem, San Diego, CA) (PMA/I, 100 ng/ml/500 ng/ml). Supernatants were centrifuged at 1400 rpm for 8 minutes at 4°C, transferred to fresh tubes, and stored at -20°C until use. Cells were washed once with 1× PBS prior to use.

**Table 1:** List of primers.

**Primer Name**	**Forward Primer**	**Reverse Primer**	**Base Primer**	**Anneal**
**TLR1**	CTATACACCAAGTTGTCAGC	GTCTCCAACTCAGTAAGGTG	220	61
**TLR2**	GTACCTGTGGGGCTCATTGT	CTGCCCTTGCAGATACCATT	191	62
**TLR3**	GATCTGTCTCATAATGGCTTG	GACAGATTCCGAATGCTTGTG	305	60
**TLR4**	ACAACCTCCCCTTCTAACC	AACTCTGGATGGGGTTTCCT	201	61
**TLR5**	CTAGCTCCTAATCCTGATG	CCATGTGAAGTCTTTGCTGC	438	59
**TLR6**	AGGTGCCTCCATTATCCTCA	GAATCCATTTGGGAAAGCAG	211	59
**TLR7**	CTCCCTGGATCTGTACACCTGTGA	CTCCCACAGAGCCTTTTCCGGAGCT	551	55
**TLR8**	GTCCTGGGGATCAAAGAGGGAAGAG	CTCTTACAGATCCGCTGCCGTAGCC	581	55
**TLR9**	TTCCCTGTAGCTGCTGTCC	ACAGCCAGTTGCAGTTCACC	207	58
**TLR10**	GGCCAGAAACTGTGGTCAAT	CTGCATCCAGGGAGATCAGT	199	61
**ER-Alpha**	TGCCAAGGAGACTCGCTACT	CTGGCGCTTGTGTTTCAAC	276	55
**ER-Beta**	TCAGCTTGTGACCTCTGTGG	TGTATGACCTGCTGCTGGAG	178	56
**PR-A**	GGTCTACCCGCCCTATCTCA	GGCTTGGCTTTCATTTGGAA	397	55
**PR-B**	GAGGGGGCAGTGGAACTCAG	AGGGGAACTGTGGCTGTCGT	293	55

### Enzyme linked immunosorbent assay (ELISA)

IL-6, IL-8, and Interferon-inducible protein 10 (IP-10) matched antibody pairs were purchased from either BioLegend (San Diego, CA) or BD Biosciences (San Diego, CA), and ELISA was performed according to manufacturer's instructions with 100 μl of cell free supernatant. IL-6 detection limit was 5 pg/ml, IL-8 was 8 pg/ml, and IP-10 was 10 pg/ml (the lowest standard used in each standard curve). Absorbance at 450 nm was read with the SPECTRAMax 190 microplate spectrophotometer, and results were analyzed by SOFTMax Pro software (Molecular Devices, Sunnyvale, CA). Sample concentrations were determined by interpolation from the standard curve. Triplicate samples for each treatment condition were analyzed in each experiment. Readings for the triplicate treatment conditions were averaged and standard deviation was calculated. Statistical analysis between treatment conditions was performed as described below. All experiments were performed a minimum of three times.

### RNA isolation

Endometrial epithelial cell lines were grown to confluence in 12 well plates and total RNA was isolated using the RNAqueous kit (Ambion, Austin, TX) per manufacturer's instructions. RNA was treated with 11 μl of Turbo DNase buffer, 2 μl of Turbo DNase, and 22 μl of Turbo DNase inactivation reagent (Ambion, Austin, TX). RNA was quantified using the RiboGreen^® ^RNA Quantitation kit (Molecular Probes, Eugene, OR), and fluorescence was measured using a 485 nm excitation filter and a 535 nm emission filter on a Fusion™ Universal Microplate Reader (Perkin-Elmer, Wellesley, MA). RNA concentrations were interpolated from a standard curve. Samples were measured in triplicate.

### End Point Reverse Transcriptase Polymerase Chain Reaction (RT-PCR) and Electrophoresis

A total of 100 ng of RNA was used to synthesize cDNA using the random primers provided in the ImProm-II™ Reverse Transcriptase System (Promega, Madison, WI). Briefly, 100 ng of RNA was denatured at 65°C for 15 minutes and immediately placed on ice for 5 minutes. RNA was combined with master mix containing 4 μl ImProm-II 5× reaction buffer, 2 μl 25 mM MgCl, 1 μl 10 mM dNTP mix, 1 μl random primers, 0.5 μl RNasin, and water. Master mix was prepared with or without AMV reverse transcriptase (RT) in a total reaction volume of 20 μl. The reaction was run using an Eppendorf^® ^Master Gradient Thermocycler (Brinkman, Westbury, NY). Polymerase chain reaction (PCR) using gene specific primers was performed using 1 μl cDNA in 25 μl total reaction volume. PCR amplification was performed using Eppendorf^® ^MasterTaq Polymerase (Brinkman, Westbury, NY). Primers for TLRs 1–10, ERα, PRB, and PRT were obtained from the literature and are listed in Table 1[25,28,29]. Primers for ERβ, also listed in Table 1, were designed using Primer3 software  (Whitehead Institute for Biomedical Research, Cambridge, MA). Loading dye (5 μl) was added to the reaction and 10 μl of sample was run on a 2% agarose 3:1 (Amresco, Solon, OH) gel to separate PCR products. Gels were stained with SYBR^® ^Green (BioWhittaker, Rockland, ME) for 45 minutes and visualized by ultraviolet transillumination at 302 nm. Digital images were obtained using a GelLogic 100 (Kodak, Rochester, NY).

### Real time RT-PCR

Real time RT-PCR was performed using cDNA synthesized as described for end point RT-PCR. cDNA was combined with primer/probe sets and Taqman^® ^Universal PCR Master Mix (Applied Biosystems, Inc., Foster City, CA). Primer/probe sets for TLR3, ERα, and hypoxanthine phophoribosyltransferase (HPRT) were purchased and designed using TaqMan Gene Expression Assays™ (Applied Biosystems, Inc., Foster City, CA). Real time assays were run on an ABI 7000 (Applied Biosystems, Inc., Foster City, CA). Samples were normalized internally using the cycle threshold (CT) of the housekeeping gene, HPRT, as follows: ΔCT = (CT TLR3 or CT ERα) - (CT HPRT). Quantitative values for ERα mRNA are demonstrated using ΔCT, where the samples with the highest quantitative level of ERα mRNA have the lowest value and the samples with the lowest quantitative level of ERα mRNA have the highest value. For analysis of quantitative TLR3 mRNA values, the mean CT of RNA from untreated cells (Norm 1) was set to a relative quantity (RQ) value of 1 using the ΔΔCT calculated as follows: ΔΔCT = mean ΔCT(Norm 1) - mean ΔCT(Norm 1) and RQ = 2^(-1*ΔΔCT)^. All other samples were compared to the mean RQ value for the untreated cells (Norm 1) using the following equation: ΔΔCT = ΔCT(sample) - mean ΔCT(Norm 1). RQ values were calculated as follows: RQ = 2^(-1*ΔΔCT)^.

### Protein Lysates and Western Blot Analysis

For preparation of protein lysates from endometrial epithelial cell lines for western blot analysis, cells were lysed by adding ice-cold RIPA lysis buffer (50 mM Tris, 150 mM NaCl, 50 mM NaF, 1 mM Na_4_P_2_O_7_·10 H_2_O, 0.1% DOC, 1.0% NP-40, 50 μl Na_3_VO_4_, and 100 μl Halt Protease Inhibitor Cocktail (Pierce Biotechnology, Inc, Rockford, IL)). After 30 min on ice with shaking, the lysates were centrifuged at 15000 × g for 10 min at 4°C. Supernatants were stored at -80°C until use. For western blot analysis, protein concentrations were determined using the BCA Protein Assay (Pierce Biotechnology, Inc, Rockford, IL). Equal amounts of unboiled protein were loaded onto a 4–20% Tris-HCl Precast Gel (BioRad) with 10 μl of sample or Blue Ranger marker (Pierce Biotechnology, Inc, Rockford, IL), and transferred to two identical 0.45 μM PVDF membranes (Millipore, Billerica, MA). The membranes were washed in Tris-buffered saline containing 0.05% Tween 20 (TBS-T) and non-specific binding sites were blocked by immersing the membrane in block reagent (1:7.5 mL Sea Block·TBS blocking buffer) (Pierce Biotechnology, Inc, Rockford, IL) for 1 hour at room temperature on a shaker. The membranes were then washed with TBS-T. One of two identical membranes was incubated overnight at 4°C with the isotype antibody in blocking buffer. The other membrane was incubated overnight at 4°C with ERα clone H-184 in blocking buffer. ERα clone H-184 was generously provided by Dr. Mark Hannink (University of Missouri-Columbia, Columbia, MO). Membranes were then washed and incubated with secondary antibody for 1 hour at room temperature. After incubation, the membranes were washed in TBS-T. Bound antibodies were detected by a chemiluminescent detection system (West Femto; Pierce Biotechnology, Inc, Rockford, IL) as recommended by the manufacturer's instructions. For exposure (10 seconds, 30 seconds, 1 min, 5 min, and up to 1 h) we used CL-XPosure™ Film (Pierce Biotechnology, Inc, Rockford, IL).

### Flow Cytometry

Cells were plated in 12 well plates and grown to confluence in CD-F12 with or without hormones. After 72 hours, cells were harvested, counted, and 0.5 × 10^6 ^cells were labeled with either PE-labeled mIgG1α hTLR3 antibody (clone TLR3.7) (eBioscience, San Diego, CA) or PE-labeled mIgG1 isotype control (BD Biosciences, San Diego, CA). For intracellular staining, cells were fixed using Cytofix/Cytoperm buffer (BD Biosciences, San Diego, CA), and all washes and incubations were performed in the saponin-containing Perm/Wash buffer (BD Biosciences, San Diego, CA). Cells were analyzed using a Fluorescence-Activated Cell Sorter (FACScan) instrument and analysis was performed using CellQuest software (BD Biosciences, San Diego, CA). Ten thousand cells were counted, and viability was determined by generating forward scatter (FSC) versus side scatter (SSC) density plots and setting the gate to exclude dead cells. Histogram plots included only gated live cells. Markers indicating TLR3 positive cells were set so that less than 10% of isotype control cells were included in the positive marker.

### Statistical analysis

Mean values for treatment conditions in triplicate were compared using one-way analysis of variance (ANOVA) and the Tukey post-hoc test to determine statistical significance. Differences were considered to be statistically significant at *P *< 0.05.

## Results

### Endometrial epithelial cell lines express TLR3 and steroid hormone receptors

**Figure 1:**
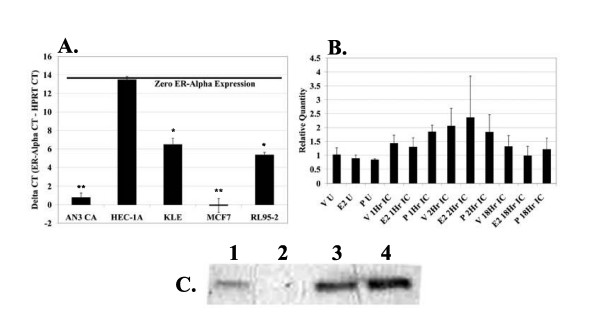
**Endometrial epithelial cells express ERα mRNA and protein**. (A) cDNA was synthesized from 100 ng of RNA isolated and subjected to quantitative Real Time RT-PCR using primer/probe sets for HPRT and ERα. Delta CT was calculated as follows: ERα CT – HPRT CT. ERα quantities ± SD from samples in triplicate are shown, with cell lines expressing the highest levels of ERα mRNA demonstrating the lowest Delta CT value. The Delta CT value representative of zero expression of ERα is shown (black bar). Statistical analysis was determined using ANOVA (*P *< 0.05). One asterisk (*) represents statistical significance as compared to bars with no asterisk. Two asterisks (**) represents statistical significance as compared to (*) and bars with no asterisk. (B) cDNA was synthesized from 100 ng of RNA isolated and subjected to quantitative Real Time RT-PCR using primer/probe sets for HPRT and ERα. Average ERα quantities (RQ values) ± SD from samples in triplicate are shown by using the housekeeping gene, HPRT, to normalize samples. (C) Protein lysates from endometrial and breast epithelial cell lines were collected and 10 μg of protein was analyzed for presence of ERα protein (67 kD) by Western Blot. Samples shown are as follows: (1) RL95-2 cells positive for ERα mRNA by endpoint RT-PCR, (2) RL95-2 cells negative for ERα mRNA by endpoint RT-PCR, (3) and (4) MCF7 positive control.

To examine the presence of TLR3, ERα, ERβ, PRA, and PRB in endometrial cell lines, mRNA was subjected to RT-PCR. The human breast adenocarcinoma cell line, MCF7, was used as a positive control for expression of mRNA coding for estrogen receptor (ER) and progesterone receptor (PR) [30-32]. We have previously demonstrated expression of TLR3 mRNA in RL95-2 cells[26]. We confirmed expression of TLR3 mRNA in the RL95-2 cells, and demonstrated expression of TLR3 mRNA in the cell lines HEC-1-A, KLE, and MCF7 (Table II). Expression of ER and/or PR has been previously demonstrated in the HEC-1-A, KLE, MCF7, and RL95-2 cells [30-34]. Receptor expression in these cell lines was confirmed and receptor expression was documented in AN3-CA cells. As summarized in Table II, many of our cell lines express detectable levels of mRNA for TLR3 and hormone receptors. We observed that the band intensity for all cell lines expressing hormone receptors was high, with intensity for the MCF7 cells being the highest. To determine if the differences observed by endpoint RT-PCR were due to quantitative differences in ERα mRNA expression, we subjected cDNA synthesized from 100 ng RNA from each of the cell lines to Real Time RT-PCR. As demonstrated in Figure 1A, our cell lines express varying quantities of ERα mRNA. The AN3-CA and MCF7 cells expressed the highest levels of ERα mRNA, with values significantly greater than values for the RL95-2, KLE, and HEC-1A cells. The RL95-2 and KLE cells expressed the second highest levels of ERα mRNA, which were significantly lower than values for the AN3-CA and MCF7 cells, but greater than values for the HEC-1A cells, which did not express ERα mRNA. Thus, the results in Figure 1A confirm the endpoint RT-PCR results shown in Table 2 and demonstrate differential expression of ERα mRNA in endometrial and breast epithelial cell lines.

**Table 2:** Epithelial cells express TLR3 and hormone receptor mRNA.

**Cell Line**	**TLR3**	**ERα**	**ERβ**	**PRA**	**PRB**
**AN3 CA**	-	+	+	+	+
**HEC-1-A**	+	-	+	-	-
**KLE**	+	+	+	+	+
**MCF7**	+	+	+	+	+
**RL95-2**	+	+	+	+	+

cDNA synthesized from 100 ng RNA was subjected to end-point PCR with the indicated primer sets (see Table 1). The endometrial epithelial cell lines, AN3-CA, HEC-1-A, KLE, and RL95-2 were scored for hormone receptor expression on the basis of a visible band at the appropriate size. The breast adenocarcinoma cell line, MCF7, served as a positive control for hormone receptor expression. Cell lines were compared to the RL95-2 cells for expression of TLR3.

To determine whether hormone treatment altered expression of ERα mRNA, we subjected the RL95-2 cell line to hormone treatment and analyzed ERα mRNA by Real Time RT-PCR. Hormone concentrations were determined by dose response as described in Methods. As demonstrated in Figure 1B, hormone treatment does not alter levels of ERα mRNA in the RL95-2 cells.

In addition to endpoint and Real Time RT-PCR, we also analyzed our cell lines for presence of ERα protein. The MCF7 breast epithelial cell line was used as a positive control (Figure 1C, lanes 3 and 4). All cell lines, with the exception of the HEC-1A cells and high passage RL95-2 cells (Figure 1C, lane 2), express ERα protein (Figure 1C and data not shown). As shown in Figure 1C, MCF7 cells (lanes 3 and 4) express a greater amount of ERα protein than the RL95-2 cells (Figure 1C, lane 1), confirming the Real-Time RT-PCR results. Additionally, RL95-2 cells lose ERα expression with high passage, which has been previously documented [37]. Due to the ability of RL95-2 cells to lose ERα expression with high passage, receptor presence had to be verified prior to experiments.

### 17β-estradiol suppresses cytokine and chemokine production in Poly I:C-stimulated RL95-2 cells

**Figure 2:**
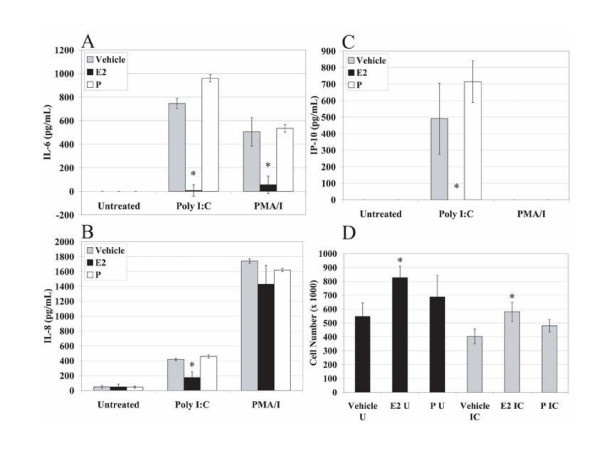
**Effects of E_2 _and P on cytokine and chemokine production and proliferation in RL95-2 cells**. RL95-2 cells were plated at 0.2 × 10^6 ^cells/well/ml and maintained in charcoal/dextran-treated DMEM/F12 (CD-F12) for 48 hours prior to experiments. Media was replaced with media containing either 10^-8 ^M E_2 _or 10^-7 ^M P as determined by dose response. Cells remained in hormone-containing media for 66 hours total and were stimulated with vehicle, Poly I:C (10 μg/ml), or PMA/I (10 ng/ml and 500 ng/ml) 18 hours prior to harvest. A total of 100 μl of cell-free supernatant was used to detect IL-6, IL-8, and IP-10 by ELISA (A, B, and C). RL95-2 cells were plated at 0.2 × 10^6 ^cells/well/ml, harvested, and counted after 18 hours of stimulation with vehicle (black bars) or Poly I:C (10 μg/ml, gray bars). The mean of three cell counts of triplicate wells counted for each time point from one experiment is shown (D). Experiments were performed using triplicate wells for each treatment condition and repeated three times. Representative data is shown. Error bars indicate standard deviation of three samples. Statistical significance (*) was determined by one-way ANOVA and the Tukey post-hoc test (*P *< 0.05).

We have previously demonstrated production of IL-6, IL-8, and the chemokine IP-10 after stimulation of RL95-2 cells with the synthetic dsRNA, Poly I:C[26]. We have also demonstrated that the response to Poly I:C in the RL95-2 cells is dependent on the expression of TLR3[26]. Since expression of TLR3 mRNA and protein was shown to be cyclically regulated[26], we performed experiments to examine whether E_2 _and P may influence TLR3 expression and/or function. A dose response was performed for E_2 _and P to determine the concentration of hormone to be used. The concentrations chosen, 10^-8 ^M E2 and 10^-7 ^M P, resulted in the greatest degree of cytokine and chemokine suppression (data not shown) and are also physiologically relevant[3,35]. Cells were stimulated with Poly I:C, PMA/I, or Poly dI:dC in triplicate. PMA/I directly activates intracellular signaling mechanisms leading to cytokine expression and was used as a positive control[26]. Poly dI:dC, a dsDNA analogue of Poly I:C, does not act as a TLR3 ligand and was used as a negative control to demonstrate the specificity of Poly I:C for TLR3[26]. Supernatants were collected and examined for production of IL-6, IL-8, and IP-10. Experiments were repeated at least three times to ensure reproducibility. RL95-2 cells treated with E_2 _showed significant suppression of IL-6, IL-8, and IP-10 protein secretion upon stimulation with Poly I:C in comparison to vehicle-treated controls and P-treated samples (Figure 2A, 2B, and 2C). Treatment with E_2 _also significantly suppressed PMA/I-induced IL-6 production, but did not significantly suppress IL-8 (Figure 2A). PMA/I treatment did not induce production of IP-10 (Figure 2C). Suppression of PMA/I response by E_2 _has been previously documented[12,36]. The significance of the suppression is notable considering the significant proliferative effect of E_2 _on RL95-2 cells as compared to vehicle-treated cells (Figure 2D). P did not induce significant proliferation of RL95-2 cells in comparison to vehicle-treated cells (Figure 2D). The response to the synthetic dsRNA, Poly I:C, was specific because stimulation with the synthetic dsDNA, Poly dI:dC, had no effect. These results suggest that E_2_, but not P, significantly suppresses proinflammatory and antiviral cytokine responses upon Poly I:C stimulation of RL95-2 cells.

### 17β-estradiol treatment does not alter TLR3 mRNA or protein expression in RL95-2 cells

**Figure 3:**
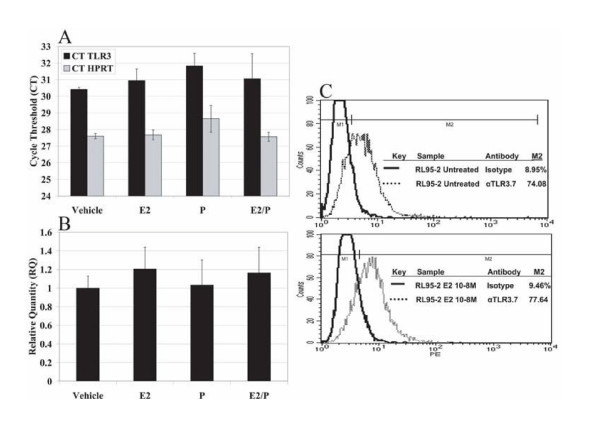
**Effects of E_2 _and P on TLR3 mRNA and protein expression**. cDNA synthesized using 100 ng of RNA from RL95-2 cells treated with 10^-8 ^M E_2 _or 10^-7 ^M P for 66 hours was used to detect expression levels of TLR3 mRNA by real-time RT-PCR. Data are shown as average cycle threshold ± SD from samples in triplicate for TLR3 and HPRT (A). Average TLR3 quantities (RQ values) ± SD from samples in triplicate are shown by using the housekeeping gene, HPRT, to normalize samples (B). RL95-2 cells were harvested after 72 hours of treatment with 10^-8 ^M E_2 _or 10^-7 ^M P and labeled with PEαTLR3.7 mAb in order to detect intracellular TLR3 protein by FACS analysis. PE-conjugated mIgG1 was used as an isotype control (C). Experiments were repeated three times. Data representative of three experiments are shown.

Previous work in our laboratory has demonstrated cyclic variation of TLR3 mRNA and protein expression in human endometrium[26], suggesting the possibility that some of the E_2 _suppression of Poly I:C-stimulated cytokine production might be due to E_2 _regulation of TLR3 expression. To examine this possibility, RNA collected in triplicate from RL95-2 cells treated with ethanol vehicle, 10^-8 ^M E_2_, or 10^-7 ^M P was used to examine TLR3 mRNA expression by real time RT-PCR. E_2 _and P did not significantly alter TLR3 mRNA expression (P > 0.05) as compared to vehicle-only controls (Figure 3A and 3B). The results in this *in vitro *model system suggest that neither E_2 _nor P directly regulates TLR3 gene transcription.

Protein expression was examined by Fluorescent-Activated Cell Sorter (FACS) analysis of RL95-2 cells treated with ethanol vehicle, 10^-8 ^M E_2_, or 10^-7 ^M P in triplicate. We had previously shown that RL95-2 cells express TLR3 intracellularly[26]. In these experiments, the intracellular expression of TLR3 in RL95-2 cells was not altered by treatment with E_2 _and P as compared to vehicle-treated controls (Figure 3C). Protein expression remained unaltered in response to E_2 _or P treatment for up to 7 days (data not shown). The lack of alteration in TLR3 mRNA and protein expression in response to E_2 _suggests that the observed suppression of cytokine production is not due to transcriptional or translational regulation of TLR3.

### Estrogen receptor is required for suppression of cytokine and chemokine production by 17β-estradiol in poly I:C-stimulated endometrial epithelial cells

**Figure 4:**
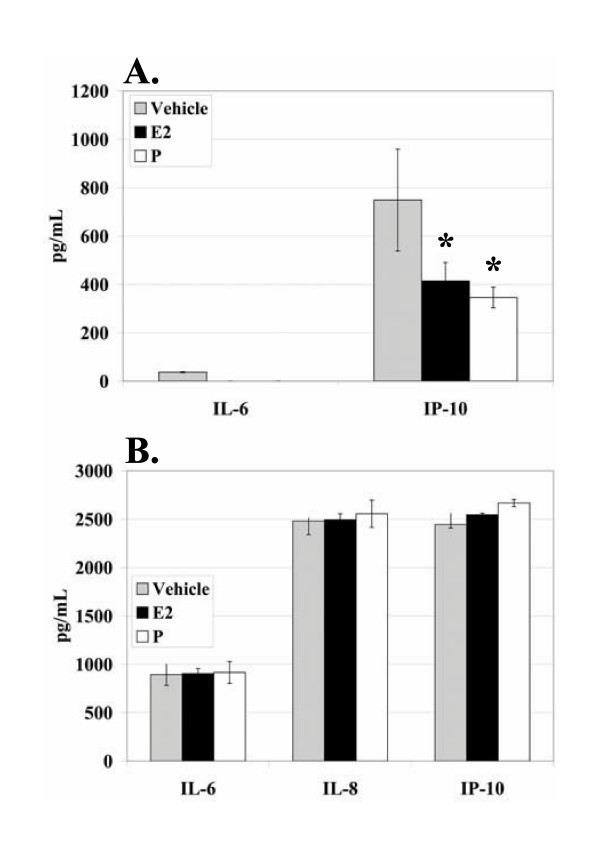
**Effects of E_2 _and P on endometrial epithelial cells expressing or not expressing hormone receptors**. KLE (A) and HEC-1-A (B) cells were plated at 0.2 × 10^6 ^cells/well/ml and maintained in CD-F12 for 48 hours prior to experiments. Media was replaced with media containing either 10^-8 ^M E_2 _or 10^-7 ^M P. Cells remained in hormone-containing media for a total of 66 hours. 18 hours prior to harvest of supernatants, cells were stimulated with the vehicle, Poly I:C (10 μg/ml), or PMA/I (10 ng/ml and 500 ng/ml). ELISA was performed with 100 μl of cell-free supernatant. Data representative of three experiments are shown. Error bars indicate standard deviation of three samples. Statistical significance (*) was determined by one-way ANOVA and the Tukey post-hoc test (*P *< 0.05).

To determine if similar effects could be observed in other endometrial epithelial cell lines, the cell lines KLE, HEC-1-A, and AN3 CA were used. As expected, the TLR3-negative, hormone receptor-positive cell line, AN3 CA, did not respond to Poly I:C (data not shown). Treatment of KLE cells with E_2 _and P resulted in a non-significant decrease in production of IL-6 and a significant decrease in production of IP-10 (Figure 4A), which was expected since KLE cells express TLR3, ER, and PR (Table II). The observed decrease in IL-6 production in the KLE cells treated with E_2 _or P was not statistically significant (*P *= 0.165) and may be due to the described defects in nuclear ER trafficking in the cell line[34]. The KLE cell line produced background levels of IL-8 too high for observation of a Poly I:C-induced effect (data not shown). A suppressive effect from hormone treatment was not observed in the HEC-1-A cells (Figure 4B), which express TLR3 and ERβ, but do not express ERα, PRA, or PRB (Table II). Our results, therefore, suggest that ERα must be present for suppression of cytokine or chemokine production. Finally, RL95-2 cells have been reported to lose ERα expression with continued passage in culture[37]. In our study, consistent with previous reports, RL95-2 cells lacking ERα did not exhibit suppressive effects after E_2 _treatment (Figure 1C and data not shown) and receptors had to be verified in the RL95-2 cells prior to each experiment to ensure reliable results. Thus, suppression of cytokine and chemokine production by E_2 _in endometrial epithelial cells may require ERα.

### Suppression of cytokine and chemokine production by estrogen in Poly I:C-stimulated RL95-2 cells is ER-dependent

**Figure 5:**
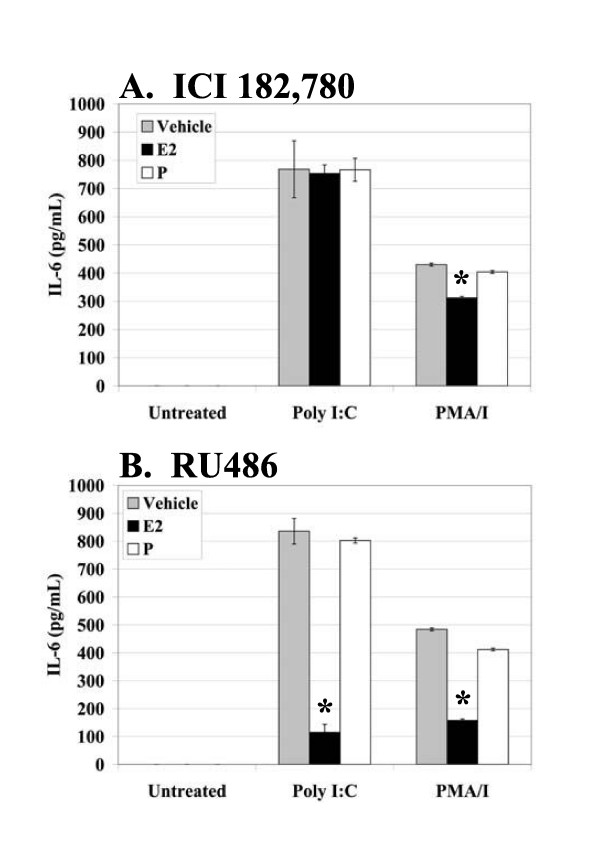
**Effects of E_2 _in RL95-2 cells are mediated by ER**. RL95-2 cells were plated at 0.2 × 10^6 ^cells/well/ml and maintained in CD-F12 for 48 hours prior to experiments. Media was replaced with media containing vehicle, 10^-6 ^M ICI 182, 780 (A), or 10^-6 ^M RU486 (B). After 2 hours, media was replaced with the indicated combinations of ethanol vehicle, 10^-8 ^M E_2_, 10^-7 ^M P, 10^-6 ^M ICI 182, 780, and 10^-6 ^M RU486. Cells remained in the indicated media for a total of 66 hours. Cells were stimulated with ethanol vehicle, Poly I:C (10 μg/ml), or PMA/I (10 ng/ml and 500 ng/ml) 18 hours prior to harvest. A total of 100 μl of cell-free supernatant was used to detect IL-6, IL-8, and IP-10 by ELISA (A, B, and data not shown). Experiments were performed using triplicate wells for each treatment condition and repeated three times. Data representative of three experiments are shown. Error bars indicate standard deviation of three samples. Statistical significance (*) was determined by one-way ANOVA and the Tukey post-hoc test (*P *< 0.05).

Since treatment with E_2 _and P did not affect TLR3 mRNA or protein expression in the RL95-2 cells (Figure 3A, 3B, and 3C), but did affect TLR3-induced cytokine and chemokine production, and hormone receptor-negative cell lines do not show a suppressive response, we postulated that the suppressive effect may be hormone receptor-dependent. To determine if the suppression of cytokine and chemokine production in RL95-2 cells was hormone receptor-dependent, we utilized the hormone receptor antagonists, ICI 182, 780 and RU486. For the no-antagonist controls, cells were treated with ethanol vehicle, E_2_, or P (representative data in Figure 2). Treatment with the ER antagonist, ICI 182, 780, restored cytokine and chemokine levels in E_2_-treated samples to those of vehicle-treated controls (Figure 5A). RU486 had no restorative effect on E_2_-treated samples, as expected (Figure 5B). Since suppression was not seen in the no-antagonist, P-treated samples, treatment with the PR antagonist, RU486, had no effect (Figure 5B). These results are not due to a suppression of cytokine and chemokine production in the vehicle-treated samples by the antagonists themselves because the antagonists caused a reduction in cell number for all antagonist-treated samples (data not shown). Thus, our results confirm that E_2 _suppresses cytokine and chemokine production in Poly I:C-stimulated RL95-2 cells in a hormone receptor-dependent manner.

### MCF7 cells express functional TLR3

**Figure 6:**
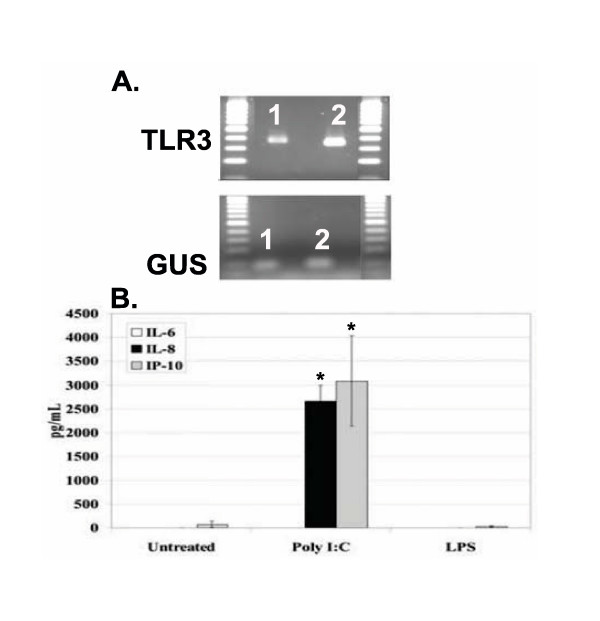
**MCF7 cells express functional TLR3**. Total RNA (100 ng) was used to generate cDNA for measurement of expression of TLR3 in MCF7 cells (Lane 1) and RL95-2 cells (Lane 2) (A). The housekeeping gene, GUS, was also included (A). MCF7 cells were plated at 0.2 × 10^6 ^cells/well/ml and stimulated with vehicle, Poly I:C (10 μg/ml), Poly dI:dC (1 μg/ml), or LPS (100 ng/ml). A total of 100 μl cell-free supernatant was used to measure IL-6, IL-8, and IP-10 by ELISA (B). Experiments were performed in triplicate and repeated three times. Data shown is representative of three experiments. Error bars indicate standard deviation of three samples per treatment condition. Statistical significance (*) was determined by one-way ANOVA and the Tukey post-hoc test (*P *< 0.05).

To determine whether the observed hormone effect was characteristic of only endometrial epithelial cells, or if the suppressive effect occurred in a different epithelial cell line, we utilized the breast epithelial adenocarcinoma, MCF7. MCF7 cells have been shown to express ER and PR and respond to E_2 _and P treatment [30-32]. MCF7 cells were examined for expression of TLR mRNA. We found that MCF7 cells express TLR2, TLR3, TLR5, and TLR6 (Table II, Figure 6A, and data not shown). For detection of TLR3 in the MCF7 cells, the RL95-2 cells were used as a positive control. The amplified band in the RL95-2 and MCF7 cells was of the appropriate size for TLR3 (Figure 6A). To determine if TLR3 expressed in these cells was functional, MCF7 cells were stimulated with Poly I:C, PMA/I, the TLR4 ligand lipopolysaccharide (LPS), or Poly dI:dC in triplicate. Supernatants were collected and examined for production of IL-6, IL-8, and IP-10. MCF7 cells, as expected, did not respond to stimulation with LPS or Poly dI:dC (Figure 6B and data not shown). MCF7 cells did produce significant amounts of IL-8 and IP-10 after stimulation with Poly I:C and IL-8 after stimulation with PMA/I (Figure 6B and data not shown). However, the MCF7 cells did not produce IL-6. Our results demonstrate that MCF7 cells express functional TLR3 and produce cytokines and chemokines upon stimulation with Poly I:C.

### 17β-estradiol and progesterone suppress cytokine and chemokine production in Poly I:C-stimulated MCF7 cells in a hormone receptor-dependent manner

**Figure 7:**
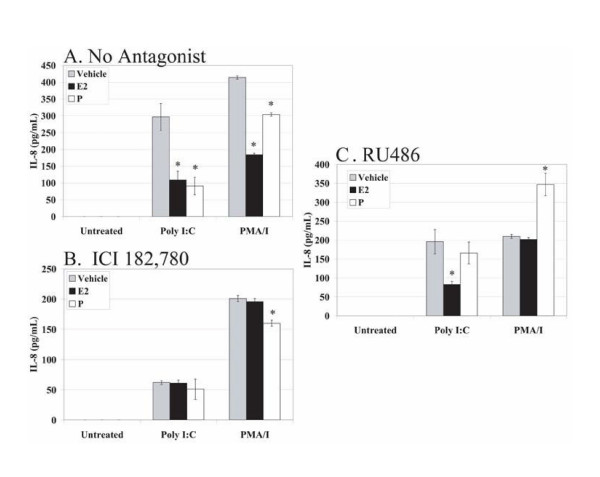
**Effects of E_2 _and P in MCF7 cells are mediated by hormone receptors**. MCF7 cells were plated at 0.2 × 10^6 ^cells/well/ml and maintained in CD-F12 for 48 hours prior to experiments. Media was replaced with media containing ethanol vehicle (A), 10^-6 ^M ICI 182, 780 (B), or 10^-6 ^M RU486 (C). After 2 hours, media was replaced with the indicated combinations of ethanol vehicle, 10^-8 ^M E_2_, 10^-7 ^M P, 10^-6 ^M ICI 182, 780, and 10^-6 ^M RU486. Cells remained in the indicated media for a total of 66 hours. Cells were stimulated with ethanol vehicle, Poly I:C (10 μg/ml), or PMA/I (10 ng/ml and 500 ng/ml) 18 hours prior to harvest. A total of 100 μl of cell-free supernatant was used to detect IL-6, IL-8, and IP-10 by ELISA (A, B, C, and data not shown). Experiments were performed in triplicate and repeated three times. Representative data are shown. Error bars indicate standard deviation of three samples. Statistical significance (*) was determined by one-way ANOVA and the Tukey post-hoc test (*P *< 0.05).

Since MCF7 cells express functional TLR3, we examined whether E_2 _and/or P suppress production of cytokines and chemokines in Poly I:C-stimulated MCF7 cells. The response to dsRNA was specific, as the MCF7 cells did not respond to stimulation with the synthetic dsDNA, Poly dI:dC. As expected, the cells did not produce IL-6 upon stimulation with Poly I:C. Cells treated with E_2 _and P exhibited a significant suppressed response to Poly I:C and PMA/I in comparison to vehicle only controls (Figure 7A). This is in direct contrast to the RL95-2 cells, in which significant cytokine and chemokine suppression when treated with P was not observed (Figure 2A, 2B, and 2C).

MCF7 cells demonstrated suppression of TLR3-induced cytokine and chemokine production in response to E_2 _and P treatment (Figure 7A) and we did not observe changes in TLR3 mRNA or protein expression in these cells (data not shown). Therefore, we wanted to determine if the suppressive responses were hormone receptor-dependent, as in the RL95-2 cells. ICI 182, 780 and RU486 have previously been shown to be effective on MCF7 cells by other investigators [38-40]. Treatment of E_2_-treated wells with ICI 182, 780 and P-treated wells with RU486 restored cytokine and chemokine levels to those of vehicle-treated controls upon Poly I:C stimulation (Figure 7B and 7C). Since treatment with antagonists resulted in a decrease in cell number (data not shown), these results are not due to the suppression of cytokine and chemokine levels by the antagonists alone. Had the antagonists caused suppression of cytokine and chemokine production, the levels of cytokine and chemokine protein in the antagonist-, hormone-treated samples would have been decreased as compared to the antagonist-, vehicle-treated samples. Thus, E_2 _and P suppress cytokine and chemokine production in Poly I:C-stimulated MCF7 cells in a hormone receptor-dependent manner as was observed in the RL95-2 cells.

## Discussion and conclusion

In this study, we provide the first demonstration that steroid hormones affect TLR function. Specifically, E_2 _modulates TLR3 function through significant suppression of Poly I:C-induced proinflammatory and antiviral cytokine and chemokine production, which we have previously shown to be dependent on TLR3 in our system[26]. The suppressive effect is not due to a decrease in the steady state levels of TLR3 mRNA or protein. However, the inhibition of function is hormone receptor-dependent, as demonstrated through the use of hormone receptor-positive and hormone receptor-negative cell lines and the hormone receptor antagonists, ICI 182, 780 and RU486.

The endometrial epithelial cell lines, AN3-CA, HEC-1-A, KLE, and RL95-2, and the breast adenocarcinoma cell line, MCF7, differentially express TLR3, ERA, ERB, PRA, and PRB mRNA. The levels of ERα mRNA in these cell lines differ quantitatively, with the MCF7 cells demonstrating the highest quantitative level of ERα mRNA and the HEC-1A cells not expressing ERα mRNA. All the cell lines utilized, with the exception of the HEC-1A cells, also express detectable levels of ERα protein. The expression of TLR3 is noteworthy because expression of TLR3 has not been documented in the HEC-1-A, KLE, or MCF7 lines. Additionally, MCF7 cells express TLR2, TLR5, and TLR6. The impact of hormones on signaling of other TLRs, including those expressed in the MCF7 and RL95-2 cells, has not been explored and will be the focus of future studies.

We found that E_2_, but not P, significantly suppresses production of both proinflammatory and antiviral cytokines in Poly I:C-stimulated RL95-2 cells. Both E_2 _and P suppress production of cytokines and chemokines in KLE and MCF7 cells. Previous reports have demonstrated suppression of IL-6 mRNA by E_2 _in cytokine-stimulated and LPS-stimulated cells[2,36], but other investigators have suggested that IL-6 production is not regulated by ovarian steroids[16,17]. The data presented in our study suggests that regulation of the TLR3 signaling pathway by E_2 _results in suppression of secreted IL-6, as well as IL-8 and IP-10. The contradictory findings in the regulation of IL-6 by E_2 _may be due to variable expression of ER in the tissues under examination[17]. Additionally, instability of hormone receptor expression in RL95-2 cells has been documented[37]. In order to address these issues, hormone receptor expression was verified prior to all experiments in this study to eliminate the possibility of incorrect observations due to variable receptor expression. Furthermore, we have observed that E_2 _suppression of Poly I:C-stimulated cytokine production in RL95-2 cells occurs only when ERα is present.

Significant P effects were observed only in the MCF7 cells. Although RT-PCR results for the RL95-2 and MCF7 cells in Table 1 demonstrate that both cell lines express PR, potential differences in quantitative PR expression may be present between the cell lines and this may account for the difference in P effects. However, other investigators have documented differential efficiency of the two PR isoforms dependent on cell context [41-43]. Thus, we speculate that differences between specific cell lines and/or differences in co-activator and co-repressor expression may underlie the differences in P action in the two cell lines.

In addition to suppression of IL-6 and IL-8 production, we demonstrate suppression of IP-10 by E_2 _in Poly I:C-stimulated RL95-2 cells. E_2 _has been shown to suppress IP-10 function in murine mammary cells[44]. However, suppression of IP-10 by E_2 _has not been previously documented in endometrial cells and tissues. Our findings are noteworthy due to the presence of TLR3, which recognizes viral dsRNA, in the endometrium and the role of IP-10 in the antiviral response. IP-10 may be important in recruitment of immune effector cells and activation of cell surface receptors essential for immune defense against viral pathogens, such as HIV, CMV, and HSV in the human endometrium[44,45]. Thus, TLR3 activation and subsequent production of IP-10 following dsRNA stimulation may be a pivotal event in modulating endometrial events.

Although our previous finding of cyclic regulation of TLR3 in the endometrium suggested potential control of TLR3 expression and function by steroid hormones, we found that E_2 _does not suppress Poly I:C-induced cytokine and chemokine production by altering TLR3 mRNA or protein expression. Thus, our findings in the *in vitro *model system suggest that E_2 _does not directly regulate TLR3 expression, but does regulate TLR3 function through interaction with ER. Previous findings suggested that maximal expression of TLR3 in the endometrium occurs in the mid and late secretory phases, a time when ERα protein in the endometrial epithelium is virtually undetectable [46-48]. Thus, the suppressive action of E_2 _on cytokine expression would serve to suppress TLR3 action at times of low TLR3 expression. Inhibition of TLR3 function would not occur during times of maximal TLR3 expression due to lack of ERα. A similar mechanism of E_2 _inhibition of expression has been proposed for the β3 integrin subunit, which is expressed on endometrial epithelium in a pattern similar to that observed for TLR3, except with the β3 integrin subunit, E_2 _inhibits expression rather than function[49].

The exact mechanism by which E_2 _regulates TLR3 function remains to be elucidated. Ray and colleagues and Galien and colleagues demonstrated an inhibition of the DNA-binding activity of the transcription factors NF-IL6 and NF-kB by the estrogen receptor, resulting in suppression of IL-6 gene expression[36,50]. However, the mechanism of IL-8 and IP-10 suppression has not been explored. IP-10 production occurs upon activation of the transcription factor, interferon regulatory factor 3 (IRF3), rather than NF-κB. Studies examining interactions of IRF3 with ER have not been performed and will be necessary to determine if the nature of the suppressive effect of E_2 _on IP-10 production is similar to that of NF-κB and ER. Our future studies will be designed to determine the mechanism by which E_2 _regulates TLR3 signaling components.

Suppression of cytokines and chemokines by E_2 _was determined to be ER-dependent in this study. Hormone effects have been shown to be hormone receptor-dependent in other studies[51]. TLR3-positive endometrial epithelial cell lines that do not express ERα, such as the HEC-1-A line, do not exhibit suppressed cytokine and chemokine production upon stimulation with Poly I:C. Additionally, treatment of TLR3-positive, ERα-positive cell lines with the ER antagonist, ICI 182, 780, restores levels of cytokine and chemokine production to vehicle only levels. All of the data presented in this study has been obtained using *in vitro *experiments with endometrial epithelial cell lines. We have previously demonstrated that TLR3 is functional in primary endometrial epithelial cells[26]. Since the response to E_2 _and P may be different in primary endometrial epithelial cells as compared to endometrial epithelial cell lines, future experiments will be performed using primary endometrial epithelial cells to address this question.

Our current and previous studies suggest a role for TLR3 in the endometrium. We have shown TLR3 expression to be cycle-dependent in primary endometrial epithelial cells and that the response to Poly I:C in our *in vitro *system requires TLR3[26]. This study demonstrates modulation of TLR3 function by the hormones of the endometrium. The cytokines and chemokines produced upon TLR3 ligation are important in normal endometrial functions and may be crucial in the pathogenesis of endometrial dysfunctions such as endometriosis [1-4]. Recognition of viral dsRNA by TLR3 and the resulting production of inflammatory and antiviral cytokines and chemokines may be pivotal in the protection of the endometrium from pathogens. The cyclic regulation of TLR3 in the endometrium may allow protection against pathogens while maintaining a system of tolerance toward the embryo and preventing extensive tissue damage from the inflammatory response. It has been suggested that development of endometrial dysfunction is characterized by an increased inflammatory environment, allowing progression of disease[6]. Thus, the influence of steroid hormones on TLR3 function, specifically the suppression of cytokines and chemokines produced upon stimulation of TLR3 by dsRNA, may be critical in the regulation, maintenance, and defense of the human endometrium.

## Authors' contributions

M.J.L. carried out the hormone studies, immunoassays, statistical analysis, and drafted the manuscript. All authors participated in the design of the study, interpretation of the results, and manuscript corrections. All authors read and approved the final manuscript.
